# Dentists’ perspectives on selective caries removal for the management of deep carious lesions in permanent teeth

**DOI:** 10.1186/s12903-025-05699-8

**Published:** 2025-03-09

**Authors:** Jennifer Kettle, Zoe Marshman, Alice Hamilton, Sarab El-Yousfi, Sarah R. Baker, Avijit Banerjee, Chris Deery, Craig R. Ramsay, David Ricketts, Janet E. Clarkson

**Affiliations:** 1 School of Clinical Dentistry, University of Sheffield, Sheffield, UK; 2 Dundee Dental Hospital & School, University of Dundee, Dundee, UK; 3 Faculty of Dentistry, Oral and Craniofacial Services, King’s College London, London, UK; 4 Health Services Research Unit, University of Aberdeen, Aberdeen, UK; 5 Dundee Dental Hospital & Research School, University of Dundee, NHS Education for Scotland, Dundee, UK

**Keywords:** Complete caries removal, Dental, Caries, Primary care, Qualitative, Randomised controlled trial, Selective caries removal, Theoretical domains framework, Minimally invasive dentistry

## Abstract

**Background:**

To explore the views of dentists participating in the Selective Caries Removal in Permanent Teeth (SCRiPT) randomised controlled clinical trial on selective caries removal versus complete or near complete caries removal for the management of deep carious lesions.

**Methods:**

Nineteen semi-structured one-to-one telephone or online video interviews were conducted with dentists involved in SCRiPT, using an interview guide informed by the Theoretical Domains Framework (TDF). Data were initially analysed deductively using a framework informed by the TDF, and subsequently using reflexive thematic analysis.

**Results:**

Three themes and 25 sub-themes were generated. Themes were ‘comfort using selective caries removal’, ‘potential value of SCRiPT’ and ‘challenges of subjectivity’. Sub-themes included six enablers and five barriers to the use of selective caries removal, as well as five contextual factors potentially impacting dentists’ decision-making. The SCRiPT trial was found to have potential value in terms of ‘overcoming uncertainty’, although perceived limitations were noted. The potential value of SCRiPT may depend on other factors, including the willingness of dentists to follow evidence from the trial (reflecting personal attributes and comfort with selective caries removal). The interviews also highlighted how caries removal is perceived as subjective and involves the application of clinical judgement to individual cases. General dental practitioners who are less comfortable with selective caries removal may not start to use this approach as defined within SCRiPT, particularly if there is a lack of strong evidence from the trial.

**Conclusions:**

Dentists’ level of comfort with selective caries removal is multi-faceted and informed by contextual factors. SCRiPT has the potential to increase acceptance of selective caries removal, but the findings may not be interpreted in this way. Future work should further explore the concept of comfort with selective caries removal, using the thematic framework outlined here to inform the design of interview topic guides.

**Trial registration:**

Trial registry: ISRCTN. Trial registration number: ISRCTN76503940. Date of Registration: 30.10.2019.

**Supplementary Information:**

The online version contains supplementary material available at 10.1186/s12903-025-05699-8.

## Background

Dental caries is a prevalent non-communicable disease that can impact negatively on quality of life [1]. When restorative interventions are required, there is a lack of consensus about how much carious tooth tissue to remove pulpally before placing a restoration in order to achieve optimal outcomes for patients [2]. Selective caries removal to soft dentine pulpally is now recommended for deep lesions in permanent teeth, over non-selective removal [3]. Deep lesions are defined as those extending into the pulpal third of the dentine [2]. However, there is a risk of bias within existing studies, and further research is required [4]. It is important to ascertain whether selective caries removal to soft dentine over the pulp will sustain tooth vitality sensibility and reduce the need for complex treatment.

The Selective Caries Removal in Permanent Teeth (SCRiPT) trial is a pragmatic, multi-centre, two-arm patient randomised controlled clinical trial taking place in the UK. The trial is comparing selective caries removal with complete or near complete caries removal for deep carious lesions in permanent posterior teeth among NHS dental attenders aged 12 and over. Selective caries removal is defined within SCRiPT as:

- Gain access to the dentine caries by removing superficial enamel or existing restoration.
- Remove caries from the periphery of the cavity to allow for good adaptation and seal to the restoration either at the enamel dentine junction or the peripheral 2 mm of dentine if the cavity margin is on root dentine.
- Remove remaining carious dentine leaving soft dentine pulpally “that deforms when an instrument is pressed into it and can be easily scooped up (e.g. with a spoon hand excavator) with little force being required”’ [2].

Complete or near complete caries removal is defined within SCRiPT as:

- Gain access to the dentine caries by removing superficial enamel or existing restoration.
- Remove caries from the periphery of the cavity to allow for good adaptation and seal to the restoration either at the enamel dentine junction or the peripheral 2 mm of dentine if the cavity margin is on root dentine.
- Remove caries to firm dentine pulpally which is “physically resistant to hand excavation and some pressure needs to be exerted through an instrument to lift it” [2].

Traditionally, ‘complete caries removal’ was used to describe non-selective removal to ‘hard’ dentine, but within SCRiPT, this term also included ‘near complete’ caries removal which has been described by the International Caries Consensus Collaboration 2016 as ‘selective removal to firm dentine’, which leaves either ‘firm’ or ‘leathery’ dentine over the pulp [3]. The term ‘complete caries removal’ was therefore changed during the SCRiPT trial to ‘complete or conventional caries removal’.

Decisions regarding caries removal are important to dentists, reflecting variation in attitudes, teaching and practice. Focus groups and interviews with dental practitioners conducted during the development of the SCRiPT protocol found variation in current behaviour and professional uncertainty around the best option for the operative management of patients with deep carious lesions [2]. Selective caries removal has not been universally adopted by dentists [5]. A survey of German dentists found that half considered only complete excavation and 70% refused selective removal in a case of a 20 year old with deep carious lesions and risk of pulp exposure [6]. Reasons given included concerns that the remaining bacteria could harm the pulp and that sealed lesions could still progress and risks to the restorations placed. Although the majority were of this view, the research identified two distinct groups with opposite attitudes towards caries removal. While more recent surveys in a number of countries have found evidence of a shift towards less invasive approaches [7, 8], such approaches have not been widely adapted in some countries, such as France and Germany [5].

Qualitative research can offer a more in-depth understanding of how general dental practitioners feel about caries removal. Dentists were interviewed in a study about selective removal in deep lesions, including the consistency of dentine respondents would leave behind [9]. At baseline, 46.6% of responding dentists would leave only hard dentine behind, 45.5% would excavate until they reached firm dentine and 5.5% would be willing to leave soft dentine. Barriers to selective removal were a lack of guidelines, discrepancies between knowledge gained at university and more recent research, lack of experience with selective removal and fear of endodontic complications and lacking a routine for applying selective removal [9].

Among undergraduate dental students, selective removal is preferred to the potential stress of pulp exposure [10]. However, at this stage there are uncertainties about how much caries to leave and concerns about secondary caries and development of pulp necrosis. Some students expressed a lack of confidence in a technique that leaves caries behind as this runs counter to traditional teaching. A sense of ‘comfort’ in this case came from not having full responsibility for these treatments [10]. While general dental practitioners have completed their training, concerns felt as an undergraduate may continue to be relevant.

Patients also have views on selective caries removal (defined as ‘leaving some caries’) and complete caries removal (defined as completely removing carious tissue) [11]. A mixed-methods study in Germany conducted focus groups with 12 adult patients, and distributed surveys to 150 patients [11]. No participants in the focus groups were aware of selective caries removal. Half thought complete caries removal was a safer, more reliable option. The risk of pulp exposure, the potential for root canal treatment, the potential need for retreatment due to recurrent caries and restorative complications were relevant factors to patients. There was an emphasis on trusting the dentist, which could put pressure on general dental practitioners making treatment decisions [11].

The aim of this paper is to explore the view of dentists involved in the SCRiPT trial on the use of selective caries removal in general dental practice.

## Methods

This study is reported in line with the consolidated criteria for reporting qualitative research (COREQ) guidelines [12] (Supplementary file 1). Ethical approval for the study was obtained as part of the ethical approval for the SCRiPT trial (North of Scotland Research Ethics Committee, 6th January 2020).

### Design

As part of the process evaluation, dental stakeholders were interviewed during the set-up phase (pre-trial interviews) and during the trial (mid-trial interviews). Interviews with health professionals within trials can be used to understand how trial- and clinical- protocols relate to prior experiences within dentistry, everyday activities in general dental practice and views on a particular issue [13].

The interviews conducted during SCRiPT were intended to explore usual care, views on the intervention prior to delivery and experiences within the trial. The interviews were guided by the TDF which was used to structure and analyse the interviews [14, 15]. The refined TDF is based on 14 domains and 84 constructs [15] (Table 1). While the domain ‘nature of behaviour’ has been removed from version 2 of TDF, this domain can be used in combination with version 2 as this allows for how relevant tasks are represented [16].

**Table 1:** TDF domains and relevance to script

TDF Domain	Definition	Example of relevance to SCRiPT
Knowledge	An awareness of the existence of something	Knowledge about different approaches to caries removal.
Skills	An ability or proficiency acquired through practice	The skill of removing caries from a deep lesion.
Social/Professional Role and Identity	A coherent set of behaviours and displayed personal qualities of an individual in a social or work setting	Understanding of caries removal in relation to the professional identity of being a dentist.
Beliefs about Capabilities	Acceptance of the truth, reality, or validity about an ability, talent, or facility that a person can put to constructive use	Confidence in ability to follow the SCRiPT protocol.
Optimism	The confidence that things will happen for the best or that desired goals will be attained	Belief that the intended goals of selective caries removal will be achieved.
Beliefs about Consequences	Acceptance of the truth, reality, or validity about outcomes of a behaviour in a given situation	Belief about patient outcomes following selective caries removal.
Reinforcement	Increasing the probability of a response by arranging a dependent relationship, or contingency, between the response and a given stimulus	Approach to caries removal reinforced by consequents within SCRiPT.
Intentions	A conscious decision to perform a behaviour or resolve to act in a certain way	Intention to use selective caries removal rather than complete or conventional caries removal.
Goals	Mental representations of outcomes or end states that an individual wants to achieve	Intended patient outcomes from using selective caries removal.
Memory, Attention and Decision Processes	The ability to retain information, focus selectively on aspects of the environment and choose between two or more alternatives	The decision making process regarding how much carious tissue to remove.
Environmental Context and Resources	Any circumstance of a person’s situation or environment that discourages or encourages the development of skills and abilities, independence, social competence, and adaptive behaviour	Aspects of general dental practice that can impact on the use of selective caries removal.
Social Influences	Those interpersonal processes that can cause individuals to change their thoughts, feelings, or behaviours	The influence of other dentists on decisions regarding selective caries removal
Emotion	A complex reaction pattern, involving experiential, behavioural, and physiological elements, by which the individual attempts to deal with a personally significant matter or event	Emotional responses to the use of selective caries removal
Behavioural Regulation	Anything aimed at managing or changing objectively observed or measured actions	Changes to behaviour regarding caries removal

TDF is a flexible framework that can be used across different settings [17]. TDF has been used in qualitative implementation research with dental professionals to understand their behaviours, motivations and cognitions, including in relation to the evidence-based management of patients with bacterial infection [18] (nine relevant domains), the management of non-cavitated proximal carious lesions [19] (ten relevant domains) and the use of a risk communication tool [20] (eight relevant domains). All domains were represented across these studies. Qualitative research should justify when not all domains are used in an individual study [21].

Interview guides for pre-trial and mid-trial interviews were designed by ZM and JK on the basis of the TDF and guidelines for conducting a process evaluation [15, 22]. Interview guides were approved by the trial team and were not piloted. The interview guide is provided (Supplementary file 2).

### Research team and reflexivity

The majority of interviews were conducted by JK (female); one interview was conducted by SE (female). JK has a PhD and a background in sociology. She has experience of interviewing and qualitative analysis. She is a research associate working independently from the trial team and is not a qualified dentist. SE is a qualified dentist with a PhD and experience of conducting qualitative research. Data analysis was supported by ZM (female), a qualified dentist with a PhD and significant experience of interviewing and qualitative analysis, and AH (female), a qualified dentist with expertise in selective caries removal, currently completing a PhD.

### Sampling and approach

Sampling was largely purposive, with some initial convenience sampling. Dentists from twelve dental practices recruited to the SCRiPT trial were contacted by email between November 2020 and February 2021 for pre-trial interviews (i.e. during the set-up phase of the trial). Four did not respond. Interviewed dentists’ gender, region and time since qualification was recorded, and as more practices were recruited to SCRiPT, dentists who had qualified recently were purposively sampled to achieve maximum variation.

The first patient was randomised to SCRiPT in June 2021. By April 2022, thirteen practices had recruited at least three patients to SCRiPT. Dentists from nine of these practices were contacted by email by the SCRiPT team for interviews for the mid-trial phase. One responded and declined to take part. In November 2022, an additional 12 dentists were contacted. These dentists were purposively sampled based on gender, region and engagement with the trial. Seven did not respond. In July 2023, 11 dentists from practices who had never recruited were contacted in order to explore reasons for non-engagement with the trial. None responded and this line of enquiry was not possible.

### Data collection

Dentists who indicated an interest in being interviewed were sent an information sheet by the interviewer (including the aim of the study) and were given the opportunity to ask questions. All participants provided informed consent by returning signed consent form prior to interview, or giving verbal consent at the time of interview. The pre-interview information included the interviewers’ background, and in the case of JK, lack of clinical training. Interviews were carried out by telephone or online video call. The length of interviews ranged from 35 to 90 min. The average length was 52 min. All interviews involved one dentist and one interviewer, and no other people were present. The interviewers had no relationships with interviewees prior to initial contact.

All interviews were audio-recorded and transcribed verbatim by an external company (Dictate2Us). Transcripts were checked for accuracy by the interviewer. Transcripts were not returned to participants for checking. Notes were made during the interviews to highlight significant points for further discussion.

### Data analysis

Analysis initially followed a framework approach, with a deductive orientation based on the TDF [23]. Data were coded and categorised using Microsoft Excel 2016 by JK. An overview of barriers and enablers to selective caries removal organised on the basis of the TDF was developed. While TDF was useful for organising the data, analytical notes on the initial overview highlighted overlaps and overarching themes that offered the potential to situate barriers and enablers to selective caries removal within SCRiPT in a wider context. On the basis of discussions between JK, ZM and AH, a set of themes in the sense of patterns of shared meaning (rather than domain summaries) were generated by JK, following the principles of reflexive thematic analysis [24, 25]. The thematic framework related to the idea of ‘comfort’ with selective caries removal. While previous qualitative research has referred to ‘comfort’ among undergraduates managing deep carious lesions [10], this was not developed as a multi-faceted concept. In this study, the term ‘comfort[able]’ emerged from the interviews with dentists and was further developed and conceptualised by JK to contextualise barriers and enablers to selective caries removal. This included identifying and naming different facets of ‘comfort’ to capture different aspects of the interview data. The thematic framework was discussed among all authors, drawing on different disciplinary and professional backgrounds, to confirm validity. Interviewed dentists did not provide feedback on these findings.

Rather than claiming saturation in the sense of ‘information redundancy’, we recognise the limitations of this concept for reflexive thematic analysis, which recognises that the meaning of these themes requires interpretation, and the themes do not ‘emerge’ from the data [26]. For instance, most dentists in this sample appeared comfortable with selective caries removal, so there are fewer examples of barriers. However, concerns and initial discomfort were important thematically, and the positive emphasis may represent sampling limitations (discussed below).

## Results

### Characteristics of participants

The final sample comprised nineteen dentists (see Table 2 – PT is used for pre-trial, MT for mid-trial). The pre-trial sample comprised eight dentists who were interviewed between November 2020 and February 2021. The mid-trial sample comprised eight dentists who were interviewed between May and June 2022 and five who were interviewed between November and December 2022. Two dentists were interviewed both pre-trial and mid-trial to explore experiences of the trial as compared to expectations.

**Table 2:** Participant details

Participant	Region	Gender	Years since qualifying	Previous trial experience	Role
PT1	Scotland	Female	4	No	Associate
PT2/MT13	Scotland	Female	33/35	Yes	Associate
PT3	Scotland	Female	16	Yes	Principal
PT4	England	Male	35	Yes	Principal
PT5/ MT1	Scotland	Male	11/13	Yes	Principal
PT6	England	Male	35	No	Principal
PT7	Scotland	Female	18	No	Associate
PT8	England	Female	5	Yes	Associate
MT2	England	Male	29	No	Principal
MT3	England	Male	45	No	Principal
MT4	England	Male	36	No	Principal
MT5	England	Female	17	No	Principal
MT6	England	Male	12	No	Associate
MT7	Scotland	Female	8	No	Associate
MT8	England	Male	30	No	Principal
MT9	England	Female	26	No	Associate
MT10	England	Male	30	Yes	Principal
MT11	England	Female	24	No	Associate
MT12	England	Female	10	No	Associate

Across the whole sample, thirteen were from England. This partly reflects the higher number of actively recruiting practices in England. Ten were female. Thirteen had no prior experience of trials. Ten were principal dentists. Dentists qualified between 4 and 45 years prior to their interview (mean: 22 years).

Interview data were allocated to all 14 TDF domains.

### Themes

Three themes and 25 sub-themes were generated through analysis of the data (Table 3). These themes and sub-themes are presented in more detail with illustrative quotes (Supplementary file 3) and illustrated with reference to contextual factors in a thematic map (Supplementary file 4). The first theme, ‘comfort using selective caries removal’ is organised into enablers, barriers and contextual factors that potentially influence dentists.

**Table 3:** Themes and sub-themes

Theme	Sub-theme
Comfort using selective caries removal	Perceived benefits of selective caries removal (enabler) Selective caries removal perceived to align with dental goals (enabler) Perceived risk of exposure from complete caries removal (enabler) Perception of patient acceptance (enabler) Ability to mitigate risk (enabler) Lack of perceived barriers (enabler) Ongoing influence of dental training (barrier) Lack of prior knowledge or experience of selective caries removal (barrier) Risk of negative perceptions of other dentists (barrier) Concern about lack of consensus (barrier) Lack of concern about the risks of complete caries removal (barrier) Existing approach (context) Approach taught at dental school (context) Attitude to changing approach (context) Access to alternative sources of knowledge (context) Goals within dentistry (context)
Potential value of SCRiPT	Overcoming uncertainty Limitations of SCRiPT to provide evidence Optimism about outcomes of selective caries removal Willingness to follow evidence from SCRiPT Importance of conclusive evidence
Challenge of subjectivity	Perceptions of caries removal as subjective Working ‘between the extremes’ Issues of interpretation Applying clinical judgement

### Comfort using selective caries removal

This theme relates to different facets of comfort: personal, intellectual, experiential, social and professional. The level of personal comfort is an emotional response to carrying out a particular procedure. In this case, it can be reflected in emotional reactions to leaving more caries than usual (for example) and may demonstrate how messages from early dental training can be internalised. However, personal comfort can change, and it may be complete caries removal that feels more uncomfortable:

‘*Oh yeah*,* as I said the first selective caries removal I did I remember thinking*,* “Oh my goodness*,* I’ve left a lot of caries in this tooth.”*’ (MT7, female, 8 years qualified, associate).

‘*I would be much softer going the other way than going back to complete caries removal. It doesn’t feel quite right*,* it doesn’t feel right anymore I suppose*.’ (MT2, male, 29 years qualified, principal).

The level of intellectual comfort relates to an understanding of the scientific rationale for a procedure; in this case, selective caries removal can make sense on the basis of engaging with research and in the context of someone’s understanding of tooth morphology, the properties of restorative materials etc. Conversely, a dentist may lack a sense of intellectual comfort if they question the underlying rationale of selective caries removal:

‘*But I would say*,* since I’ve been teaching in* [name of university] *over the last four or five years*,* I was much more practicing towards selective caries removal without maybe going quite as far as that. Just because of the way that undergraduates are taught these days*. […] *So*,* yeah*,* so*,* you know*,* you can’t avoid but to see that all the time when you’re exposed to it*.’ (MT2, male, 29 years qualified, principal).

*‘I think if you’ve got too larger proportion of the restoration seated on soft dentine*,* particularly for some materials like composite or amalgam. I don’t think that it will give you as good longevity. I don’t think the fillings would last as well*.’ (MT6, male, 12 years qualified, associate).

The level of experiential comfort develops from personal experience with a procedure. In this study, previous experience with selective caries removal could be reassuring in terms of perceived success and patient outcomes, or dentists could feel they lacked this experience.

Social comfort reflects the wider context in which a procedure is carried out and is shaped by perceptions of the views of others and wider acceptability. In this case, dentists are concerned patients and other professionals may view selective caries removal as a mistake or evidence of negligence, rather than a valid choice, which has potential implications for patients (if unnecessary restorations are carried out). As discussed below, SCRiPT has the potential to address dentist’s ‘worry’ by improving awareness and thus improve social comfort:

‘*There’ll be a lot of dentists that aren’t trained in selective caries removal or know nothing about it whatsoever. So*,* to them leaving decay behind in a tooth is alien. You know so I think a fear would arise from*,* if we were to leave decay behind then the patient was to go and see somebody who was like that*,* not trained in it*,* that they could start giving the patient the wrong idea that the previous dentist has been negligent. I think that’s the main fear that we’d have.*’ (MT10, male, 30 years qualified, principal).

*‘I always worry that if I leave caries that people are going to get re-restorations that they don’t need so I thought bringing this to the forefront and maybe making people more aware of it would help with that.*’ (MT7, female, 8 years qualified, associate).

The level of professional comfort refers to factors that mitigate against any medico-legal risks of a particular procedure such as documenting the decision to use selective caries removal in patient notes. One dentist had started to give patients a piece of paper recording the study tooth (although obviously not the result of randomisation). She explained this in terms of recognising patients’ limited ability to remember and explain prior treatment to a new dentist:

‘*And then if*,* you know*,* that there’s something different that’s been done for this tooth*,* so whoever sees you is able to understand that the dentist has treated this tooth*,* but there’s something that the patient is not able to explain. And that is why*,* you know*,* if they want to know more about it*,* or ask anything*,* they could contact us. So yes*,* the patient is aware. So that’s not a problem.*’ (MT11, female, 24 years qualified, associate).

Understanding comfort as multi-faceted is useful for understanding the potential enablers and barriers to the use of selective caries removal outside SCRiPT. Dentists who had already accepted the rationale of selective caries removal, and/or had experience of using this approach successfully with patients, presented as more comfortable in their interviews. Dentists who were less familiar with selective caries removal appeared more uncertain, for example, highlighting a range of potential concerns in pre-trial interviews, or retrospectively in mid-trial interviews. For instance, MT11 noted her initial concerns, which had changed during the SCRiPT trial:

‘*I’m not sure if the selective helps to contain the caries long term. So*,* I may be wrong. I mean*,* my thought process is*, ***was ****I would say. But now it has changed since I’ve joined the SCRiPT. So*,* initially I would think that just leaving some caries behind would mean there’s a high chance of caries progression. So*,* there’s a chance of the patient coming back with pain or problems.*’ (MT11, female, 24 years qualified, associate).

The main barriers related to a lack of intellectual comfort (due to the ongoing influence of dental education that emphasised complete caries removal) and a lack of social comfort, reflecting how other dentists would perceive visible caries on a radiograph. Both of these contributed to the more emotional, and less tangible, personal comfort. Dentists used the hypothetical scenario of a patient visiting another dentist. However, when questioned, they acknowledged this was unlikely (for example, due to having ‘loyal’ patients, and due to professional comfort from documentation in patient notes). This concern may therefore emphasise a perceived lack of consensus among dentists, and the potential for one’s intentions to be misinterpreted, with implications for patient perceptions of a dentists’ competence and professional identity.

In the context of SCRiPT, some dentists expressed a personal preference for selective caries removal regardless of what evidence the trial might generate:

‘*Yes*,* in case if there is not much of significant difference involved*,* the approach is then I would possibly still carry on with the selective caries removal approach*,* in case*,* because I believe that that would be the right approach*.’ (MT5, female, 17 years qualified, principal).

‘*We still prefer selective caries removal over complete caries removal. Has it changed my mind about that being part of SCRIPT? No*,* not really. We still*,* you know*,* it’s our preferred method of choice*.’ (MT10, male, 30 years qualified, principal).

These examples can be understood in terms of the concept of comfort outlined above. For instance, MT5 referred to experiential knowledge of selective caries removal prior to SCRiPT, and MT10 taught minimal invasive dentistry and was convinced by the rationale. An existing sense of comfort with selective caries removal can affect how SCRiPT is viewed and engaged with, with implications for recruitment, for example.

A range of factors were noted that could impact on dentists’ personal comfort with selective caries removal, including: the existing approach prior to SCRiPT and whether this involved some version of selective caries removal; the length of time qualified and the approach a dentist was taught; a dentist’s attitude to changing their approach (e.g. willingness to access further training and engage with research); a dentist’s access to alternative sources of knowledge (for example, opportunities to teach undergraduates) and; goals within dentistry, and whether these include goals aligned with a minimally invasive approach.

### Potential value of SCRiPT

In relation to the previous theme, SCRiPT was seen to have potential value as a source of trusted knowledge, that could contribute to the consensus on selective caries removal, and thus professional comfort. Dentists who were comfortable with this approach ‘hope[d]’ the trial supports their existing views, and were optimistic about patient outcomes.

*‘I hope that the study is actually going to prove that the selective caries removal is the better approach as opposed to complete caries removal.*’ (MT9, female, 26 years qualified, associate).

However, there were examples of dentists who questioned the value of SCRiPT, for example, noting that dentists in the trial are likely to take extra care when using this approach, and that the findings may be less applicable to usual practice outside the trial.

SCRiPT was also seen to have value in providing an opportunity for dentists to gain experience of selective caries removal. Through involvement in the trial, interviewed dentists noted they had developed intellectual and/or experiential comfort (for instance, through reading literature provided within the trial or having the opportunity to treat patients resulting in positive outcomes):

‘*So initially I thought I would try it a bit and I’ve had no problem*,* no patients coming back*,* so then I’m using it more and more and so now I use it almost entirely now all the time*.’ (MT13, female, 35 years qualified, associate).

While SCRiPT was seen to have potential value as a source of evidence on selective caries removal, these findings indicate that perceptions of this value may vary among dentists. In particular, the facets of comfort outlined above, and the range of contextual factors that affect dentists’ sense of comfort (i.e. dental education, time qualified, attitude to changing approach, access to alternative sources of knowledge and goals within dentistry), may act to moderate the willingness to follow the evidence from SCRiPT, for example if there is a lack of intellectual and professional comfort:

‘*Certainly*,* one of them [dental colleague] would need concrete evidence-base to move forward on them*. […] *Yes*,* they try to follow the rules and regs* [understand as regulations] *to the tee which is great*,* and they’d be uncomfortable*,* I think*,* doing selective caries removal unless it was proven to be a better performing restoration in the end*.’ (MT8, 30 years qualified, principal).

The importance of conclusive, clear-cut evidence was highlighted if dentists are to change from an approach with which they feel comfortable; dentists may be more willing to follow evidence supporting a pre-existing attitude.

### Challenge of subjectivity

Interviewed dentists emphasised how caries removal was subjective, and in relation to the previous theme, it was noted that this could be a potential limitation to SCRiPT, as dentists may interpret the protocol in different ways. One participant who had previously worked as a nurse commented:

*‘I think in dentistry things are very different. If people are from different universities or they’ve had different*,* looked at different evidence*,* or sometimes the evidence is not all clear*,* so*,* they do things differently. And sometimes you get the same result*,* but there’s lots of different ways of getting there. Yeah*,* I think probably it is quite unique to dentistry*.’ (MT12, female, 10 years qualified, associate).

Several dentists referred to their usual approach as in some way ‘between’ complete caries removal and selective caries removal, and these discussions acknowledged the challenges of adhering to particular definitions in a consistent way. The language used suggested that, despite the training, ‘complete caries removal’ may be interpreted as nonselective removal to hard dentine, rather than selective removal to firm dentine. For instance:

‘*I would say the norm is*,* nearly complete caries removal*,* but just leave*,* leaving the*,* the… leaving carious dentine over the pulp to avoid exposure. I would have said the norm is*,* is like halfway in between the two sides of the trial really’* (MT6, male, 12 years qualified, associate).

While the interviews found that dentists generally described themselves as ‘confident’ in their ability to follow the protocol, there were indications that the way in which the protocol was interpreted may vary, with implications for fidelity.

More generally, given the perception that caries removal is subjective, dentists emphasised their skill at applying clinical judgement, a competence that develops with experience and reflection:

‘*It’s developed and you kind of see what things work and I’ve seen lots of restorations that I’ve left a little bit of caries over the pulpal area and it hasn’t caused any issues so that kind of possibly reinforces you could do that again and you can check it on bitewings subsequently. Now*,* I’ve been in the practice for five years*,* I’ve re-bitewinged all my patients twice so like I can now see my restorations I’ve done and that I think makes you a better practitioner because you reflect on the margins of them and the contact points on them. So that*,* I would say you develop with that*.’ (MT7, female, 8 years qualified, associate).

Decisions to selectively leave carious dentine over the pulpal floor in the cavity preparation prior to restoration, whether this follows the SCRIPT protocol for selective caries removal or a less conservative approach with leathery or firm carious dentine over the pulp, can be understood as an example of this clinical judgement. However, dentists may feel some sense of personal and social discomfort with this approach, despite intellectual, experiential and professional comfort, particularly when there was a degree of internalised social pressure and concerns about a lack of consensus within the profession.

## Discussion

This paper has explored the perspectives of dentists on selective caries and complete or conventional caries removal in the context of the SCRiPT trial. The interviews explored dentists’ views on caries removal prior to recruitment to SCRiPT and experiences of caries removal within the trial. Analysis identified themes of comfort of selective removal, potential value of SCRiPT and the challenge of subjectivity. This paper cannot comment on fidelity data from SCRiPT. Nonetheless, it is important to highlight this particular challenge in relation to understanding the eventual trial outcomes.

The term ‘comfort’ emerged from the interviews, and was developed as an original concept by the authors. This included identifying and naming different facets that captured the different aspects discussed in the qualitative interviews. Elsewhere, the comfort zone is defined as ‘a behavioural state within which a person operates in an anxiety-neutral condition, using a limited set of behaviours to deliver a steady level of performance, usually without a sense of risk’ [27]. This has been used in the context of comfort with clinical practice, in a way that emphasises emotional experiences [28]. The term ‘comfort’ has been used elsewhere in relation to dentists’ attitudes to their work, which recognises the potential to be ‘uncomfortable’ with a particular situation (e.g. the provision of treatment during the COVID-19 pandemic) [29]. The theme of ‘being outside one’s comfort zone’ has also been used to refer to a lack of familiarity and ‘wariness’ among dentists, relating to clinical challenges and difficulties with particular groups of patients in the context of stressors and coping mechanisms in primary care dentistry more generally [30]. There is scope for further work exploring ‘comfort’ that draws on this literature, and develops ‘comfort’ as a multi-faceted concept in relation to particular areas of dental work.

The notion of comfort provides a useful conceptual tool to make sense of the ways in which dentists discuss selective caries removal, and attitudes within the context of an RCT. While some individual dentists appeared comfortable with the idea of selective caries removal, others seemed more uncertain (for example, experiencing a lack of professional comfort due to concerns about the threat of medico-legal action). The idea of distinct themes among dentists with regard to knowledge of, attitudes towards and behavioural approaches to caries removal has been noted elsewhere [6]. Further research with dentists who identify as being less comfortable with selective caries removal would be useful for understanding the range of attitudes to this approach.

The interviews demonstrated how attitudes to selective caries removal could change, both prior to SCRiPT and during the trial. Similarly, dentists involved in a trial that compared conventional and biological approaches for the management of carious lesions in primary teeth found that meanings of particular approaches had the potential to shift over time, from ‘unfamiliar’ to ‘familiar’ and ‘effective’ (in the case of biological) [13]. Nevertheless, dentists also employed experiential and interpersonal knowledge when treating patients, and appeared to value feeling ‘comfortable’ with the approach used (although this concept was not operationalised in this earlier study). More generally, clinicians who are not in individual equipoise when recruiting for a trial may not feel a sense of ‘comfort’, which can impact on recruitment and retention of sites [31–34]. The importance of feeling a sense of ‘comfort’, and more specifically, what it means to feel comfortable, would benefit from further research.

In relation to previous research on selective caries removal, there are similarities to themes identified by Jeggle and colleagues [9]. For example, the barrier of discrepancies between dental training is similar to the barrier of ‘ongoing influence of dental training’ and the facilitator of knowledge about selective caries removal as evidence based and proven was similar to ‘accepting perceived benefits of selective caries removal). Similarly, dentists continuing to show concern about using a technique that leaves caries because this is different from traditional teaching demonstrates that this feeling may continue from being an undergraduate [10], although this study has shown how a sense of comfort can develop. Enablers and barriers to the use of selective caries removal identified in other studies can be conceptualised using our notion of ‘comfort’, for example in terms of professional, intellectual and experiential comfort, all of which contribute to a less tangible sense of personal comfort. Other aspects of our analysis, while not listed as themes, are evident in the selected quotes (for instance, references to caries removal as a ‘gut feeling’ reflects a perception of the subjectivity of caries removal) [9]. Nevertheless, there are differences; for example, while dentists in Germany, the United States and New Zealand compare their practice to other dentists [19], it is not clear that they are specifically concerned about the potential negative perceptions of any other dentists a patient may see in the future, which was notable in our research. It would be useful to further explore potential barriers and enablers to selective caries removal, and one approach would be to use interview guides developed on the basis of the TDF and the thematic framework outlined above.

### Strengths and limitations

This study contributes to existing literature on dental professionals’ perspectives on selective caries removal, focusing on two nations in the UK. Using qualitative research allows for a detailed understanding of dentists’ attitudes to selective caries removal. The context of the SCRiPT trial provides a unique situation to explore this topic, which is of recognised significance to dentists [2]. The conceptualisation of a multi-faceted notion of dentists’ ‘comfort’ with a clinical procedure makes a novel contribution to the existing literature in dentistry and similar healthcare decision situations.

Nevertheless, there are some limitations to this study. All interviews were conducted with dentists recruiting for the SCRiPT trial, and therefore the sample comprised dentists who were at least willing to take part in a trial involving selective caries removal. While dentists highlighted the potential for misinterpretation of radiographs by other dentists, there was limited potential to explore this from the perspective of dentists who do not accept the rationale for SCRiPT. Although the researchers made attempts to interview dentists from practices that were involved in SCRiPT but not recruiting, in order to explore barriers experienced by these practices, these attempts were not successful. This is an acknowledged risk of embedded qualitative research and future research may benefit from exploring other forms of data collection and recruitment of ‘non-standard’ participants [33]. Nevertheless, there were examples of dentists who, while signed up for SCRiPT, were less convinced about the potential benefits of selective caries removal than others. Other dentists also acknowledged how their own perspective had changed over time, including during the course of the trial. These varied perspectives have allowed this paper to highlight the importance of comfort as a significant theme.

In addition, a longitudinal approach, involving end-of-trial interviews, would have allowed dentists to further reflect on their perspectives on complete caries removal and selective caries removal within SCRiPT, and with regard to how they anticipated working outside of the trial. Future qualitative studies within trials may benefit from a longitudinal approach, which should be planned as part of the research design. However, this also necessitates addressing how qualitative research within clinical trials is funded to ensure a researcher is available for interviewing and analysis.

All but one interview were conducted by a social scientist without clinical experience. Given the clinical subject matter, an experienced clinician may have responded to what the dentists said in a different way, establishing more technical detail. However, there are benefits to a non-clinical interviewer, who may be less likely to introduce bias in the sense of guided questions due to preconceived ideas either way. Furthermore, the findings were discussed within a research team with prior experience of the TDF and clinical experience of caries removal, which helped to clarify the overall significance.

The topic guide was designed on the basis of the TDF, and as reported above, data was initially coded in terms of barriers and enablers to selective caries removal in relation to TDF constructs. Re-analysing data on the basis of a central multi-faceted concept of ‘comfort’ provides a helpful way to understand how dentists within SCRiPT discussed selective caries removal. As outlined above, the thematic framework developed here can inform future research. Nonetheless, the authors acknowledge the limitations in not planning for a reflexive, inductive approach. Firstly, it is likely the interview guide would have been different had it not been informed by the TDF, and piloting the original interview guide would have been a useful opportunity to identify limitations of this approach. Secondly, as an alternative, planning to use a grounded theory approach and designing the research on this basis with iterative data collection and analysis, may have led to richer data. In relation to this, theoretical sampling would have required actively sampling to look for deviant cases. As noted above, this research was limited as all dentists interviewed were willing to take part in SCRiPT, and as the theoretical framework was developed following the interviews, it has not been tested with dentists who may hold different attitudes to selective caries removal and may have challenged this central concept.

### Implications for future research and practice

This paper therefore offers a *potential* thematic framework that requires further research outside the context of the SCRiPT trial. This study offers perspectives on dentists enrolled in a particular trial. The potential for negative views from other dentists emerged as the main barrier to the use of selective caries removal outside SCRiPT. Dentists anticipated lacking social comfort, although this could be mitigated to some extent through practical measures that contributed to a sense of professional comfort. Future research could usefully explore this further outside the context of SCRiPT as part of research on attitudes to caries removal more widely.

## Conclusions

Dentists within the SCRiPT trial identified barriers and enablers to their use of selective caries removal outside the trial, and contextual factors and moderators that could influence dentists’ decision-making. Enablers to the use of selective caries removal contributed to a sense of comfort. Dentists’ level of comfort with selective caries removal was multi-faceted and informed by contextual factors. SCRiPT has a potential value to increase comfort with selective caries removal, but the findings may not be interpreted in this way. Future work should further explore the concept of comfort with selective caries removal, using the thematic framework outlined here to inform the design of interview topic guides.

## Electronic supplementary material

Below is the link to the electronic supplementary material.

Supplementary file 1: COREQ checklist. A COREQ checklist for this paper. (.doc)

Supplementary file 2: Interview guide. The interview guide used with dentists. (.doc)

Supplementary file 3: Themes and sub-themes. Details of the thematic framework with illustrative quotations. (.doc)

Supplementary file 4: Thematic map. A figure mapping out the thematic framework. (.pdf)

## Data Availability

Interviewees did not consent to data sharing and therefore we are unable to share full interview transcripts. Copies of other materials (information leaflet, consent form, interview schedule) are available from the corresponding author.

## Abbreviations

COREQ: Consolidated criteria for reporting qualitative research
NHS: National Health Service
PhD: Doctor of Philosophy
SCRiPT: Selective Caries Removal in Permanent Teeth trial
TDF: Theoretical Domains Framework
