# Supplementary material for: Dentists’ perspectives on selective caries removal for the management of deep carious lesions in permanent teeth
Source: BMC Oral Health. 2025 Mar 9;25:362. doi: 10.1186/s12903-025-05699-8 (PMC11892160; doi:10.1186/s12903-025-05699-8)
Supplement: Supplementary file 1 — COREQ checklist. A COREQ checklist for this paper. (.doc) [file 12903_2025_5699_MOESM1_ESM.docx]

**Supplementary File 1: Consolidated criteria for reporting qualitative studies (COREQ) checklist**

| **Item** | **Response** | **Included in manuscript** |
| --- | --- | --- |
| Interviewer/facilitator | The first author was the researcher who conducted the majority of the interviews. One interview was conducted by a co-author on the paper | Yes (p.7) |
| Credentials | Both interviewers have a PhD. One researcher supporting the analysis has a PhD and the other is currently completing one. | Yes (p.7) |
| Occupation | The main interviewer is a research associate independent of the trial team and without clinical training. One interview was conducted by a research associate independent of the trial team with clinical training. | Yes (p.7) |
| Gender | The interviewers were female. | Yes (p.7) |
| Experience and training | The interviewers have experience of conducting qualitative research. They were supported by other researchers, one of whom also has experience of conducting qualitative research. | Yes (pp.7) |
| Relationship established | The interviewers had no relationships with the participants before the study commenced. | Yes (p.8) |
| Participant knowledge of the researcher | The interviewers’ aim in conducting the research was disclosed to participants (to find out about the implementation of SCRiPT (whether as anticipated or in actuality, depending when the interview was conducted), and dentists’ use of complete caries removal and selective caries removal more generally). | Yes (p. 8) |
| Interviewer characteristics | Details relating to the interviewers’ occupation (see above) were disclosed to participants. | Yes (p.8) |
| Methodological orientation and theory | The research was informed by the Theoretical Domains Framework. | Yes (p.6) |
| Sampling | Participants were purposively sampled with some initial convenience sampling. | Yes (p.7) |
| Method of approach | Participants were contacted by email by the SCRiPT team. | Yes (p.7) |
| Sample size | 19 participants were interviewed for the study (two on two occasions). | Yes (p.9) |
| Non-participation | Dentists who were approached and who did not respond, or who did not take part in an interview, are listed in the paper. | Yes (p.8) |
| Setting of data collection | Data was collected in telephone interviews and in online video interviews. | Yes (p.8) |
| Presence of non-participants | No non-participants were present during the interviews. | Yes (p.8) |
| Description of sample | The stage of the trial, and the gender, length of time since qualifying, region and role of participants in the trial are included in a table. | Yes (Table 2, pp.30-31) |
| Interview guide | Interview guides were used, based on the TDF and process evaluation guidance. These were approved by the trial team and not pilot tested. | Yes (pp.6-7) |
| Repeat interviews | Two repeat interviews were carried out. | Yes (pp.9-10) |
| Audio/visual recording | All interviews were audio recorded. | Yes (p.8) |
| Field notes | Notes were made during the interviews to highlight significant points for further discussion. | Yes (p.8) |
| Duration | Interviews averaged 52 minutes and ranged from 35 to 90 minutes. | Yes (p.8) |
| Data saturation | Data saturation is discussed. Due to the limitations of the study it is not possible to say data saturation has been achieved. | Yes (p.9) |
| Transcripts returned | Transcripts were not returned to participants for comment. | Yes (p.8) |
| Number of data coders | Data were coded by the first author only. | Yes (p.8) |
| Description of the coding tree | The original coding tree is not described in depth. | Yes (p.8) |
| Derivation of themes | Initial themes and revision of themes are discussed. | Yes (p.9) |
| Software | Microsoft Excel was used to manage the data. | Yes (p.8) |
| Participant checking | Interviewed participants did not provide feedback on these findings. | Yes (p.9) |
| Quotations presented | Quotations are presented to illustrate findings. Quotations are identified by a participant reference number including stage of the trial, gender, length of time since qualifying and role within the dental practice. Region can be established from Table 2. | Yes (e.g. p.11, Table 2 (pp. 30-31) |
| Data and findings consistent | We believe there is consistency between the data presented and the findings. The findings are set out with examples, and example quotes are provided for all themes and sub-themes | Yes (pp. 10-17, see also supplementary file 3). |
| Clarity of major themes | Major themes are listed in a table and discussed in the manuscript. | Yes (pp. 10-17, see also supplementary file 3) |
| Clarity of minor themes | Minor themes are listed in a table and discussed in the manuscript. | Yes (pp. 10-17, see also supplementary file 3) |
