# Supplementary material for: Dentists’ perspectives on selective caries removal for the management of deep carious lesions in permanent teeth
Source: BMC Oral Health. 2025 Mar 9;25:362. doi: 10.1186/s12903-025-05699-8 (PMC11892160; doi:10.1186/s12903-025-05699-8)
Supplement: Supplementary file 2 — Interview guide. The interview guide used with dentists. (.doc) [file 12903_2025_5699_MOESM2_ESM.docx]

**Supplementary File 2: Interview Guide**

**SCRIPT Process Evaluation:**

**Qualitative Interviews: Stakeholder Topic Guide**

**Introduction**

- The purpose of these interviews is to find out about how the intervention will be implemented in practice at the start of the study and then at a later point, how dental professionals have responded to SCRIPT and about any other factors that are relevant in terms of what you do day-to-day and how this has been affected by COVID-19. I’d like to start the interview by discussing what you normally do, and then we’ll talk about SCRIPT. I’d also like to talk more generally about using complete caries removal and selective caries removal.
- I’d also like to make it clear I’m a social researcher and I don’t have a clinical background. So at some points I might ask you to clarify something or for a bit more detail.
- Interview
  - The interview will last as long as you wish, but on average interviews last 20 – 40 minutes.
  - I will be recording the interview so I can focus on what you’re saying. I may also make some notes, for example to remind me to return to something interesting that you’ve said.
  - The interview is not a test; there are no right or wrong answers. You are the expert and I want to hear about your experience.
  - You don’t have to talk about anything you don’t want to. If I ask a question you don’t want to answer, tell me, and we’ll move on to another topic. If you want to have a break at any point, we can also temporarily stop the interview.
  - Your participation is voluntary, if you change your mind you can completely stop the interview at any point.
- Confidentiality
  - Your answers will be private. I’ll use the responses from all the interviews I’m carrying out so I can explain to the researchers carrying out the study what stakeholders think, but all the responses will be anonymised.

Do you have any questions for me before we start?

**Interview (pre-trial)**

[Turn on recorder]

Context

Can you introduce yourself and tell me a bit about your background? (date when qualified, brief professional history, further qualifications, experience of restorative techniques, experience of taking part in research)

We’re interested in looking at how this intervention works in practice, so we need to understand how dental practices work and the possibility of changing what you do.

Could you start by telling me about how this practice usually works on a day-to-day basis? (Size of practice, profile of patients, balance of responsibilities), thinking about mainly before the pandemic.

As measures relating to COVID-19 are still in place, could you briefly tell me about how your day-to-day practice has been affected? We’ll come back to this topic later in the interview

Usual practice

I’d like to start by getting an idea of what you usually do to manage patients, thinking about before the pandemic.

As you know, this study is about patients aged 12 and over with deep caries in an adult pre-molar or molar tooth. Could you tell me about how you would usually treat a patient who presented with deep caries?

- Probe
  - Removal of caries – how much
  - Restoration – approach
  - Monitoring success of restoration– e.g. more general use of radiographs etc.

Decisions about treatment

We’re interested in the different ways dentists manage patients with deep caries, and how you make decisions about what to do.

[Refer back to previous answer and establish the range of techniques used]

- Probe
  - Differences in treatment between patients – reasons for this

What do you take into account when making a decision?

What do you think is important when deciding how to manage a patient with deep caries?

[If applicable, referring back to previous answer]

Are there any situations when you would use selective caries removal?

Do you have any concerns about using selective caries removal?

Knowledge of techniques

We’re also interested in understanding knowledge about complete caries removal and selective caries removal. What did you know about these two techniques before you got involved in SCRIPT?

[Make sure participants cover knowledge of both]

- Probe
  - What covered during dental education?
  - Any additional training?
  - Information from research papers, conference presentations etc.
  - Knowledge about colleagues’ practices – other dentists

Opinions about techniques

Do you have any concerns about patient outcomes from complete caries removal? [advantages, disadvantages]

What about selective caries removal?

- Probe
  - Reasons for concerns
  - All patients, or particular patients?

Confidence using techniques

We’re also interested in understanding how confident dentists feel about their ability to remove caries. How would you describe your confidence at removing caries?

Is there anything you find difficult about removing caries?

[If previous experience of selective caries removal]

How did you find removing a selected amount of caries?

- Probe
  - Determining amount to remove

SCRIPT

I’d like to move on to talk about your thoughts on the SCRIPT study. Could you tell me how you came to be involved in SCRIPT?

- Probe
  - Reasons for interest
  - Thoughts on intervention
  - Support from practice

How did you see SCRIPT fitting into your day-to-day practice, before dental practices were closed?

Training in the intervention

As part of the SCRIPT study, I believe you took part in a face-to-face training course to prepare you for delivering the intervention. Could you tell me about that?

- Probe
  - Opinions of training for intervention
  - What was particularly helpful
  - Anything that could be improved

If training was offered more widely on selective caries removal, would you recommend it to a colleague?

- Probe
  - Reasons for recommending
  - Reasons for not recommending

Following the training, how do you feel about following the protocol?

Did you have any concerns about delivering the intervention, thinking back to before practices closed?

Moving on to thinking about how COVID-19 has affected your management of patients

Has your approach to managing patients with deep caries been affected by COVID-19? If so, in what ways?

- Probe
  - Capacity
  - Working arrangements
  - Use of aerosol generating procedures
  - PPE
  - Perceived attitudes of patients to treatment

How might it affect your management of patients with deep caries in the future as the effect of the pandemic changes?

How has it affected your thoughts on how the SCRIPT study will run?

**Ending the interview**

Thank participant

Reiterate confidentiality

Confirm willingness to be contacted for a second interview

**Interview (mid-trial)**

[If this is first interview]

Context

Can you introduce yourself and tell me a bit about your background? (date when qualified, brief professional history, further qualifications, experience of restorative techniques, experience of taking part in research)

We’re interested in looking at how this intervention works in practice, so we need to understand how dental practices work and the possibility of changing what you do.

Could you start by telling me about how this practice works on a day-to-day basis? (Size of practice, profile of patients, balance of responsibilities)

In brief, how has your practice been affected by COVID-19?

Usual practice

I’d like to start by getting an idea of what you usually do to manage patients outside of SCRIPT.

As you know, this study is about patients aged 12 and over with deep caries in an adult pre-molar or molar tooth. Could you tell me about how you would usually treat a patient who presented with deep caries?

- Probe
  - Removal of caries – how much
  - Restoration – approach
  - Monitoring success of restoration– e.g. more general use of radiographs etc.

Has your approach to managing patients outside of SCRIPT been affected by COVID-19? If so, in what ways?

SCRIPT

I’d like to move on to talk about your experiences in the SCRIPT study. Could you tell me how you came to be involved in SCRIPT?

- Probe
  - Reasons for interest
  - Thoughts on intervention
  - Support from practice

[If this is second interview]

In your previous interview, you talked to me about your usual practice and your thoughts on the intervention before you started recruiting.

[Review first interview prior to second interview, and familiarise self with summary of key points – potentially add questions relating to specific expectations/perceptions as compared to reality]

Both

Recruitment and reach

I’d like to talk a bit about recruitment to the study and how patients responded to the possibility of selective caries removal. How have you found recruiting patients?

Were there any difficulties with recruitment?

How did you find explaining the study?

Did patients express any concerns about the study?

How did you deal with that?

We’re interested in eligible patients who decline to participate, could you tell me a bit about any patients you spoke to who decided not to take part?

- Probe
  - Reasons given by patients
  - Any particular groups of patients

[Also follow-up any reference to COVID-19, if not mentioned, probe for any effects]

Restoration

[Relate to previous discussion regarding experience of SCR/CCR – e.g. may need to rephrase question if participant has extensive experience of selective caries removal]

How did you find carrying out selective caries removal in practice?

How did you find following the protocol regarding selective caries removal?

Were there any challenges determining how much caries to remove in any individual cases?

How did you tend to restore the teeth?

Protocol

Were there any issues following the protocol more generally?

How did you find remembering the protocol when talking to patients?

Is there anything that you think could have been improved about the protocol?

Were there any occasions where you didn’t follow the protocol? Why not?

Did you put anything in place to make it easier to follow the protocol?

[Also follow up any references to COVID-19, probe for any effects of COVID-19 on following the protocol]

Training in the intervention

As part of the SCRIPT study, I believe you took part in some form of training to prepare you for delivering this intervention.

- Probe
  - Which form of training – face-to-face, online

Now you’ve recruited for SCRIPT and delivered the intervention, could you tell me what you think about the training you received?

- - Opinions of training for intervention
  - What was particularly helpful
  - Anything that could be improved

If training was offered more widely on selective caries removal, would you recommend it to a colleague?

- Probe
  - Reasons for recommending
  - Reasons for not recommending

Views following SCRIPT

Now you’ve taken part in SCRIPT, how do you feel about the two treatment options?

Do you have any concerns about using selective caries removal as a technique in the future?

Usual practice

Since taking part in SCRIPT, have you made any changes to the way you treat patients with deep caries who aren’t part of the study?

When do you tend to use complete caries removal outside of the study?

What about selective caries removal?

Thinking about the practice where you work, what support is in place for using selective caries removal?

Is there any additional support that would help you to use this approach more often?

Is there anything that makes it difficult for you to use selective caries removal as a technique?

- Probe
  - Any need for further training?

[Also follow up any references to COVID-19, probe for any impact on usual practice]

Context

What do you know about your colleagues approach to managing patients with caries?

You’ve said you think [x] about selective caries removal, to what extent do you think your views are shared by your colleagues?

Do you feel any pressure to manage patients with caries in a particular way?

[Also follow up any references to COVID-19, probe for any impact on pressure to manage caries]

Practices following results of SCRIPT

I’d like to explore a hypothetical situation. If SCRIPT was to find that selective caries removal was more effective, how do you think that would influence what you do normally?

[Depends on answer – using selective caries removal more often, not changing practice]

In this situation, what would make it easier for you to use selective caries removal with more of your patients?

- Probe
  - Views of patients regarding consequences
  - Own views regarding consequences
  - Practical issues in practice
  - Issues relating to ability

SCRIPT – overall views

Bearing in mind everything we’ve talked about, how did you find taking part in SCRIPT overall?

Is there anything you think could have been improved about this study?

Finally, given the aim of this research is to evaluate how delivering this intervention works in dental practices, is there anything else I should know?

**Ending the interview**

Thank participant

Reiterate confidentiality
