# Supplementary material for: Dentists’ perspectives on selective caries removal for the management of deep carious lesions in permanent teeth
Source: BMC Oral Health. 2025 Mar 9;25:362. doi: 10.1186/s12903-025-05699-8 (PMC11892160; doi:10.1186/s12903-025-05699-8)
Supplement: Supplementary file 3 — Themes and sub-themes. Details of the thematic framework with illustrative quotations. (.doc) [file 12903_2025_5699_MOESM3_ESM.docx]

**Supplementary File 3: Themes and sub-themes**

| **Theme 1: Comfort using selective caries removal** | | |
| --- | --- | --- |
| ***Enablers*** | | |
| **Sub-theme** | **Definition/Finding** | **Quotes** |
| Perceived benefits of selective caries removal. | Dentists may change their practice relating to caries removal as a result of attending conferences, engaging with research or taking part in undergraduate teaching. Dentists gain knowledge from personal experience of selective caries removal, and the ability to review patients over time, which influences their beliefs about the consequences of using selective caries removal. Other potential benefits for dentists included selective caries removal as a quicker procedure, and a reduced need for root treatment. | ‘*And I do keep up with recent updates in dentistry, what’s going on so I have picked up snippets along the line about leaving non-infected caries which is what I'm doing*.’ (PT2, female, 33 years qualified, associate)  ‘*But I would say, since I’ve been teaching in [university] over the last four or five years, I was much more practicing towards selective caries removal without maybe going quite as far as that. Just because of the way that undergraduates are taught these days*. […] *I mean, there’s a big emphasis* *now on the teachings of Banerjee in King’s and so… So, yeah, so, you know, you can’t avoid but to see that all the time when you’re exposed to it*.’ (MT2, male, 29 years qualified, principal)  ‘*I have some cases actually on more than three years follow up where I've done selected caries removal and the tooth has remained vital on deep carious lesions*.’ (PT8, female, 5 years qualified, associate) |
| Selective caries removal perceived to align with dental goals | Dentists describe goals such as preserving tooth tissue and protecting the nerve, and note how selective caries removal aligns with this. Goals can relate to a particular understanding of what dentistry should be. | ‘*Well, I think, we’re sort of conditioned* *now, or we are being conditioned to try and preserve as much tooth surface as possible. Whereas historically, you know, we were trying to produce, trying to prepare teeth and crowns and all this sort of thing and taking a lot of tooth surface away. So, it’s only logical that, you know, if you’re trying to preserve as much tooth surface as possible then selective caries goes hand-in-hand with that really.*’ (MT2, male, 29 years qualified, principal)  ‘*So there’s occasions where patients have already, they’re already aware, okay, we’re removing some of the decay, if we go any deeper then we’re going to go into the nerve, or we’re going to go subgingival and then most likely that we’ll need to take the tooth out. And I give them the option and say, “Look, do you want me to remove some of the decay?” And it gives them a chance of keeping the tooth for that little bit longer*.’ (MT1, male, 13 years qualified, principal) |
| Perceived risk of exposure from complete caries removal. | When discussing usual care, dentists highlight their concern about the risk of exposure in deep carious lesions, and their decision-making to leave caries in order to avoid this. This does not necessarily constitute selective caries removal as per the SCRiPT protocol, but reflects an understanding of the risks of removing all caries. There were also examples of emotional responses to using complete caries removal within SCRiPT when a dentist had moved away from this approach. | ‘*If it’s a large cavity when the hole is getting bigger and there's a risk of going into the nerve, I leave some of the caries*.’ (PT2, female, 33 years qualified, associate)  ‘*So, with the complete caries removal, unfortunately, there’s always that risk of exposing the pulp and further treatment need straight away and obviously complications associated with those*.’ (MT9, female, 26 years qualified, associate)  ‘*Yes, it was a bit a nerve-wracking, it was like a bit scary, just to be a bit, you know, that just in case it did cause more damage to the tooth.’* (MT5, female, 17 years qualified, principal) |
| Perception of patient acceptance. | Dentists interviewed during the trial generally did not see patient acceptability of selective caries removal as a significant barrier. There was an emphasis on trust, and the ability to reassure patients through explanation. | ‘*A lot of patients also think it might give them a chance of keeping the tooth if you’re not drilling it right down to removing every bit of decay*.’ (MT1, male, 13 years qualified, principal)  ‘*Patients trust you. I mean I think that's the key, you know, there's very few patients that will not trust you, or they won’t be there if they didn't trust you. So, I don't think I have any issues with patients, patients generally take on board what you're saying.*’ (MT10, male, 30 years qualified, principal)  *I'm like, “often I will leave a bit of decay anyway so this would just be slightly more than what I would leave. I’ll make sure it's well-sealed regardless of what filling goes in there”. So, in that way, I think they're reassured.* (MT7, female, 8 years qualified, associate) |
| Ability to mitigate risk | Despite the barrier of ‘risk of negative perceptions of other dentists’, the interviews indicated how medico-legal risks could be mitigated, e.g. through recording decisions in patient notes. | ‘*But if the patient forgets about it, and there's a question, then the clinical notes are there to back up what, you know, what was explained, what was done. So, I don't think that should be a major problem*.’ (MT8, male, 30 years qualified, principal)  ‘*For me personally, no, not a problem. I don't think it's an issue. I think we, you know, as long as our notes are okay, we're generally quite good*.’ (MT10, male, 30 years qualified, principal) |
| Lack of perceived barriers. | When considering the potential future use of selective caries removal, dentists note a lack of barriers that would impact on their intentions to use selective caries removal. | ‘*I don’t think that’s an issue at all really from our point of view. I don’t think it increases the time to do the job, it doesn’t add complicated situations to the job*.’ (MT8, male, 30 years qualified, principal)  ‘*And it's not a new treatment, but it's a technique that could be easily, you know, incorporated into your daily practice. So, I think it would be welcomed as a procedure to be incorporated into routine dental care.*’ (MT11, female, 24 years qualified, associate) |
| ***Barriers*** | | |
| Ongoing influence of dental training. | Dental training that emphasises complete caries removal continues to be relevant to dentists’ current approach, and can be understood as internalised social pressure to remove more decay. This can be reflected in a lack of personal comfort with selective caries removal (at least initially). | ‘*You tend to stick with how you were trained unless you go to courses and it’s embedded and there’s evidence to do something els*e.’ (PT2, female, 33 years qualified, associate)  ‘*I just remember being in cons* *clinics, and kind of it just being drilled that you have to remove all of the decay. Like there’s a bit of decay left here, you must remove that, you’ve got to remove that decay. So, yeah, I think that’s just stuck with me, I’m like “I’ve got to go and remove all the decay, I can’t leave anything behind*.”’ (PT3, female, 16 years qualified, principal)  ‘*Didn’t feel comfortable leaving the sort of very soft stuff that we’re expected to leave with selective caries removal now*.’ (MT2, male, 29 years qualified, principal) |
| Lack of prior knowledge or experience of selective caries removal. | Dentists who lack experiential knowledge may be less personally comfortable with selective caries removal. | *‘I think part of what can make me uncomfortable with it would be if I’ve not been on a practice long enough to review these patients myself*.’ (PT1, female, 4 years qualified, associate)  ‘*But obviously there’s a slight mind set change with the selective caries removal because you got to leave a lot more in than I would normally feel comfortable doing. That’s the difference*.’ (MT4, male, 36 years qualified, principal) |
| Risk of negative perceptions of other dentists. | When discussing dentists’ concerns about using selective caries removal, the most frequent response focused on the perception of other dentists. Dentists highlighted how visible caries on a radiograph might be perceived as poor practice by another dentist, and result in unnecessary treatment for patients. Interviews also commented on the related consequence of patient complaints and formal investigations. | ‘*If somebody else is looking at what I’ve done and isn’t, say, happy with the techniques themselves and thinks I’ve left decay behind accidentally or carelessly, or if the patient moves practice and goes somewhere else, and I think it’s, that probably makes me quite wary*.’ (PT1, female, 4 years qualified, associate)  ‘*You don’t want them to have x-rays with other, other dentists, and the dentist go, “Oh, the last dentist has left decay under your filling. You need a new filling in it.” Because, a), it makes the patient think that the work you’ve done is wrong. And you wouldn’t want the patient to have to undergo another procedure unnecessarily because of what you’ve done. So, you could say there is a pressure for radiographically a tooth to not look carious under your restoration because you don’t want to…you could say to look negligent or look…make it look like the patient’s going to need further work in the future.*’ (MT6, male, 12 years qualified, associate)  ‘*I think what worries a lot of people is that someone could go down the road to see, you know, another dentist, they do some bitewings, and it looks like you've left caries everywhere, and actually, you've carefully considered it and sealed it in, and there's a reason for it, and you've documented it, but that dentist doesn't know. And then if they tell the patient that you've left caries everywhere, then you could end up being investigated through no fault of your own when you've actually tried to do something that's valid*.’ (MT12, female, 10 years qualified, associate) |
| Concern about lack of consensus. | Interviews demonstrated how dentists could be concerned about a lack of consensus regarding selective caries removal, with the idea dentists may not necessarily be aware of the relevant evidence. | ‘*This idea of selective caries removal and, and leaving some caries in, it is something that’s been…there’s not much research about it, I don’t think, or evidence base behind it.*’ (MT6, male, 12 years qualified, associate)  ‘*A lot of people don’t want to learn new things, they’re happy with doing things the old way’* (MT13, female, 35 years qualified, associate)  ‘*I think this is the key, you know, people, that you can leave university and never touch it, never worry about tooth decay or updating your knowledge ever again, because it's not part of mandatory CPD and I think that's the problem*.’ (MT10, male, 30 years qualified, principal) |
| Lack of concern about the risks of complete caries removal. | Dentists were not particularly concerned about potential risks of complete caries removal and did not necessarily emphasis a tension for change. | ‘*Conventional caries removal does work. You just, you know, when you, you know, you've got people that are coming back and they’re stable, you know. I've got a stable list, hopefully, because I'm doing my job properly and taking caries away that needs to come away, if that makes sense.*’ (MT12, female, 10 years qualified, associate)  ‘*I haven’t any problems with that. I mean I've been in this game a long time. A couple of deep fillings, I've had to root fill. They come back in pain so I’ve had to root fill but only a couple so, no, I've had no comeback problem*.’ (PT2, female, 33 years qualified, associate) |
| ***Context*** | | |
| Existing approach | There were a range of existing approaches among the dentists interviewed, and examples of variation in different circumstances. | ‘*We would tend to take more caries away, ideally taking most of the caries away, just leaving some slight staining at the bottom of the cavity.*’ (PT4, male, 35 years qualified, principal)  *‘And on the whole, I would try and clear the margins, try not to go too deep. […] I tend to be as minimalist as possible*.’ (PT6, male, 35 years qualified, principal)  ‘*It varies on…I would have said the cavity that you’re preparing. Because if you’re…if you’ve got a MO or…I don’t know if you understand this term, but an MO or a DO cavity, I think you’ve got to remove more caries, get…because you need, you need a stronger restoration.* (MT6, male, 12 years qualified, associate) |
| Approach taught at dental school | As recorded in Table 2, dentists had been qualified different lengths of time, and there were reports of different approaches being taught when they were at dental school. | ‘*Full caries removal. So when I graduated it was all. “You need to remove all the caries, you can’t leave any soft caries behind, you have to remove it all*.”’ (PT5, male, 11 years qualified, principal  ‘*So, we’re mainly, I recall, was taught about the stepwise technique, so going in and removing just the outer edges and temporising things with a filling and then going back into the tooth sort of six months down the line and changing the restoration.’* (PT1, female, 4 years qualified, associate |
| Attitude to changing approach | While this cannot be formally assessed for each participant, this was acknowledged to be a relevant factor. Participants felt some dentists are more willing to change approach than others. There were examples of dentists who had changed approach prior to their involvement in SCRiPT. | ‘*Well, I think it’s always a personal choice. Some people are more open to change and new information, new evidence as such and they're happy to learn throughout their career. And there are other people who whatever they learn and works for them, it's what is right and why change things if it worked for decades? So, I think it's always a personal decision and the character of the person*.’ (MT9, female, 26 years qualified, associate)  ‘*Probably when I graduated, probably my goal would be to remove all of the caries. Whereas now, I think it’s a bit more of a grey area. I wouldn’t really commit to removing all of the caries. I would be a bit, yeah, a bit more kind of selective – in perhaps just having the clinical experience to know where the pulp lies, taking into account the age of the patient, size of the pulp, what is likely to happen. So, I would be looking at perhaps leaving more of a layer of infected tooth tissue.*’ (PT7, female, 18 years qualified, associate) |
| Access to alternative sources of knowledge | Some dentists had previous experiences of research involving biological techniques (e.g. Hall crowns), and others were involved in teaching at dental schools or supervising foundation dentists, which provided access to alternative sources of knowledge prior to SCRiPT. Knowledge can also come from discussions with colleagues, in the sense of informal peer review. This could be contrasted with a lack of access to such knowledge. | ‘*But I would say, since I’ve been teaching in [university] over the last four or five years, I was much more practicing towards selective caries removal without maybe going quite as far as that. Just because of the way that undergraduates are taught these days*.’ (MT2, male, 29 years qualified, principal)  ‘*Some dentists are just stuck in the 1980’s. Because if you think about it, a dentist my age* [PT2 qualified 33 years ago] *who’s working on his own in a single surgery practice, there's no input from other dentists coming in, he’s doing what he was trained to do. He doesn’t know anything else to do. So, he's still treating as he was treating in the ‘80s which a lot of things have changed*.’ (PT2, female, 33 years qualified, associate) |
| Goals within dentistry | The interviews provided examples of goals within dentistry, such as protecting the nerve and working in the best interest of the patient. The goal of protecting the nerve and generally being more ‘minimalist’ arguably reflects a shift within dentistry. It was noted that other dentists may be influenced by other factors, for example, financial motivations, that may impact on their attitude to selective caries removal. | ‘*But most of the time, particularly with deep caries, my objective is really to stabilise and try and protect the nerve. Because once you start doing root treatments on molars, you… the tooth is lost, really, yeah, you know. I think, you know, you’re committing a, for example, you know, a six or a five, you’ve got deep decay and starting to do root treatment on them, you know, it’s… we have to consider some sort of crown. Long-term prognosis, it has to be very valid, so, on the whole, I tend to be as minimalist as possible, possibly advising that they may need to be revisited*.’ (PT6, male, 35 years qualified, principal)  ‘*It’s always that, because we want to do the best for the patient, the best for their tooth. So, the pressure is always there to do the right thing*.’ (MT5, female, 17 years qualified, principal) |
| **Theme: Potential value of SCRiPT** | | |
| **Sub-theme** | **Definition/Finding** | **Quotes** |
| Overcoming uncertainty | SCRiPT is seen as a potentially valuable source of knowledge that could reinforce individual dentists’ positive views of selective caries removal and contribute to a wider understanding within dentistry. The idea of SCRiPT contributing to a ‘consensus’ was noted, and dentists valued the idea of having a conclusive result that could be widely shared. SCRiPT also provides the opportunity to gain experience using selective caries removal, and dentists reported feeling confident after participating in the training, and while dentists initially reported being ‘careful’ and taking more time over selective caries removal, their confidence could improve with experience. Within SCRiPT, consent within the trial contributed to professional comfort. | ‘*I think the impact will be that it’s more acceptable to leave some caries in the tooth. And I think it will sway dentists to not need to remove as much caries, and that they can afford to leave some caries over the pulp. I think it will probably be a good outcome, really*.’ (MT6, male, 12 years qualified, associate)  ‘*It will be great if it showed like one was far superior and then everyone could know about it and that would all be good.’* (MT7, female, 8 years qualified, associate)  ‘*After the training, I think it was February I went and did the training, on a Saturday morning, and the training was great. So after that I was quite confident*.’ (PT3, female, 16 years qualified, principal)  ‘*I never knew when to stop that selective caries removal initially, so, that, so, I was too careful doing it because when I did the typodont tooth, it always end up ─ ended up in complete caries removal rather than selective. So, that's why I had to just take a step back and be careful on what I do. So, but now I don't feel a difference. I'm doing it the same, you know, within the same time frame. Yes*.’ (MT11, female, 24 years qualified, associate) |
| Limitations of SCRiPT to provide evidence. | Some limitations were noted with regard to the potential knowledge gained from SCRiPT. For instance, there were concerns that the follow-up period covered by SCRiPT (three years) was not long enough to gain knowledge in relation to patient outcomes. It was also suggested that dentists may be taking extra care when using selective caries removal within SCRiPT, and that this may not extrapolate to the outside world. | **‘***But I think the thing is that the period of SCRiPT isn’t very long enough, isn’t it? It only ran for three years. Three years, ideally it should be a lot longer than three years, isn’t it? Because sometimes, you get…sometimes, the restored tooth doesn’t have symptoms until many years later, well beyond three years*.’ (MT3, male, 45 years qualified, principal)  ‘*I mean I think anybody who’s doing the trial is going to be probably trying very hard to do it very well, so I think these are all going to be restorations placed very carefully and by skilled operators and I don’t know whether that’s going to extrapolate into the real world as well as you might want it to. No, that’s my concern, is that sort of operator bias*.’ (MT4, male, 36 years qualified, principal) |
| Optimism about outcomes of selective caries removal | Dentist may be optimistic due to lack of problems/symptoms among patients treated using selective caries removal which influences beliefs in the consequences of this approach. | ‘*I feel that the selective caries removal is going to be more successful*.’ (MT8, male, 30 years qualified, principal)  ‘*Now the patients whom I have done selective treatment, they are asymptomatic. So, that gives me that confidence to understand that this treatment works in my hands. And what I thought previously, that this could progress into an infection is not the case. Yes, so, seeing results first-hand, although it's a short period I must say*.’ (MT11, female, 24 years qualified, associate) |
| Willingness to follow evidence from SCRiPT | Dentists discussed their willingness to follow the findings of the SCRiPT trial when making decisions about selective caries removal. There were examples of being more or less willing depending on the findings | ‘*Once the results of SCRiPT come out, I think I will follow whatever the recommended pathway is.*’ (MT7, female, 8 years qualified, associate)  ‘*I’m pretty open-minded you know, I try to keep learning about better outcomes for the patients, absolutely, what it’s all about. So if this SCRIPT trial proves that one way is better than the other, then that’s the way we’ll go forward*.’ (MT8, male, 30 years qualified, principal)  *‘I would have no hesitation following selective caries removal as a protocol going forward’* versus ‘*I suppose reluctantly—I would be much softer going the other way than going back to complete caries removal*.’ (MT2, male, 29 years quailed, principal) |
| Importance of conclusive evidence | In the interviews there were references to SCRiPT needing to show one approach is ‘significantly better’, or be ‘statistically important’ or ‘really prove[s]’ that an approach works. The importance of conclusive evidence may relate to existing approaches and attitudes to change; dentists seemed less willing to change from an approach they were comfortable with without definitive evidence. | ‘*Certainly, one of them [dental colleague] would need concrete evidence-base to move forward on them*. [...] *Yes, they try to follow the rules and regs* [understand as regulations] *to the tee which is great, and they’d be uncomfortable, I think, doing selective caries removal unless it was proven to be a better performing restoration in the end*.’ (MT8, male, 30 years qualified, principal)  ‘*I don’t think it will make a whole lot of difference unless the results are very clear cut one way or the other. I’ll still deal with each case as it comes*.’ (MT4, male, 36 years qualified, principal) |
| **Challenge of subjectivity** | | |
| **Sub-theme** | **Definition/Finding** | **Quotes** |
| Perception of caries removal as subjective | Caries removal is presented as subjective, in the context of dentistry more generally. The importance of ‘feel’ was highlighted in these interviews, and was seen to be relevant to decisions about caries removal. This can be perceived as an issue with for SCRiPT, in terms of the need to develop clear definitions for the protocol. | ‘*There's always…there's always that challenge of how much caries to remove because we don't really know exactly what the definitive cut-off point is because it's all subjective, it's gone by feel. It's not even gone by colour anymore, it’s really gone by feel*.’ (MT10, male, 30 years qualified, principal)  *‘I do think caries removal in general is subjective. And every different dentist would do something differently, even if you ask them to do the same thing*.’ (MT12, female, 10 years qualified, associate)  ‘*I don’t envy you trying to come up with a protocol that makes sense out of it because as I say it’s a matter of degree, and I know we’ve got calibration with those teeth and all the rest of it but actually when you’re dealing with a real carious tooth, sometimes it’s a little bit grey at the edges*.’ (MT4, male, 36 years qualified, principal) |
| Working ‘between the extremes’. | When comparing their usual approach to the protocol, dentists reported not adhering completely to either approach, as being ‘between’ the options within SCRiPT. | *‘So, I would probably say that my technique would be somewhere between the extremes of SCRiPT, between selective caries removal and complete caries removal. In that, I wouldn’t leave soft caries as I have been in selective caries removal through SCRiPT*. *So I’m kind of halfway house really*.’ (MT2, male, 29 years qualified, principal)  ‘*I would say probably I’m already, you know, kind of in between’* (PT7, female, 18 years qualified, associate) |
| Issues of interpretation | It was noted that it can be more challenging to follow the protocol for selective caries removal, particularly for secondary caries. While this wasn’t necessarily presented as a barrier that would stop a dentist deciding to use selective caries removal, there were indications that further training could help dentists to decide how to correctly use selective caries removal in these situations. | ‘*I’ve found it very challenging to do the selective caries removal when it’s secondary caries. Because if you’ve got a restoration in place, I find it very difficult to figure out where the margins are. I mean, obviously, when there’s a cavity there and there’s caries there, it’s very clear, you know, there’s caries, there’s tooth. When you’ve got a restoration there as well that you’ve got to try and work with, I find that quite difficult to establish what I’m supposed to do.* (MT2, male, 29 years qualified, principal)  ‘*I think sometimes the one I would say is more difficult if you're treating secondary caries over primary caries. Like all the demo ones we’ve done and all the practices we’ve done are all primary caries. But if you're treating secondary caries, sometimes, like you obviously have to remove the whole restoration. And then sometimes, there's not a lot of… like the caries are deep under the restoration but there’s not a lot of caries. So, you think if you're doing selective caries removal, you think, “Oh, normally, I’d leave a lot more caries than this because I wouldn’t start touching that bit but it wasn’t caries, it was restoration so I had to remove it”*.’ (MT7, female, 8 years qualified, associate) |
| Applying clinical judgement | Applying clinical judgement is a recognised skill for dentists, and in relation to caries removal, it is important to respond to the actual situation. While there may be recommendations from SCRiPT, dentists note that individual situations will require clinical judgement, which may or may not involve selective caries removal. | ‘*One thing that I would say is dentistry is a very practical job that the more you do, the more confidence you get with it, the more, the more teeth you drill whether you’re doing fillings, root canal work, the more experience you get, you could say the better you get clinically and practically. And you just get like a feeling for where…what’s a happy medium, how much caries to remove. You’ve just got that, that sixth sense really, the more you do*.’ (MT6, male, 12 years qualified, associate)  ‘*I think I probably feel the same that I did before. I think for some people it's the right thing to do, but I don't feel like it's the right thing to do for everybody*.’ (MT12, female, 10 years qualified, associate) |
