# Supplementary figures and images for: Dentists’ perspectives on selective caries removal for the management of deep carious lesions in permanent teeth
Source: BMC Oral Health. 2025 Mar 9;25:362. doi: 10.1186/s12903-025-05699-8 (PMC11892160; doi:10.1186/s12903-025-05699-8)

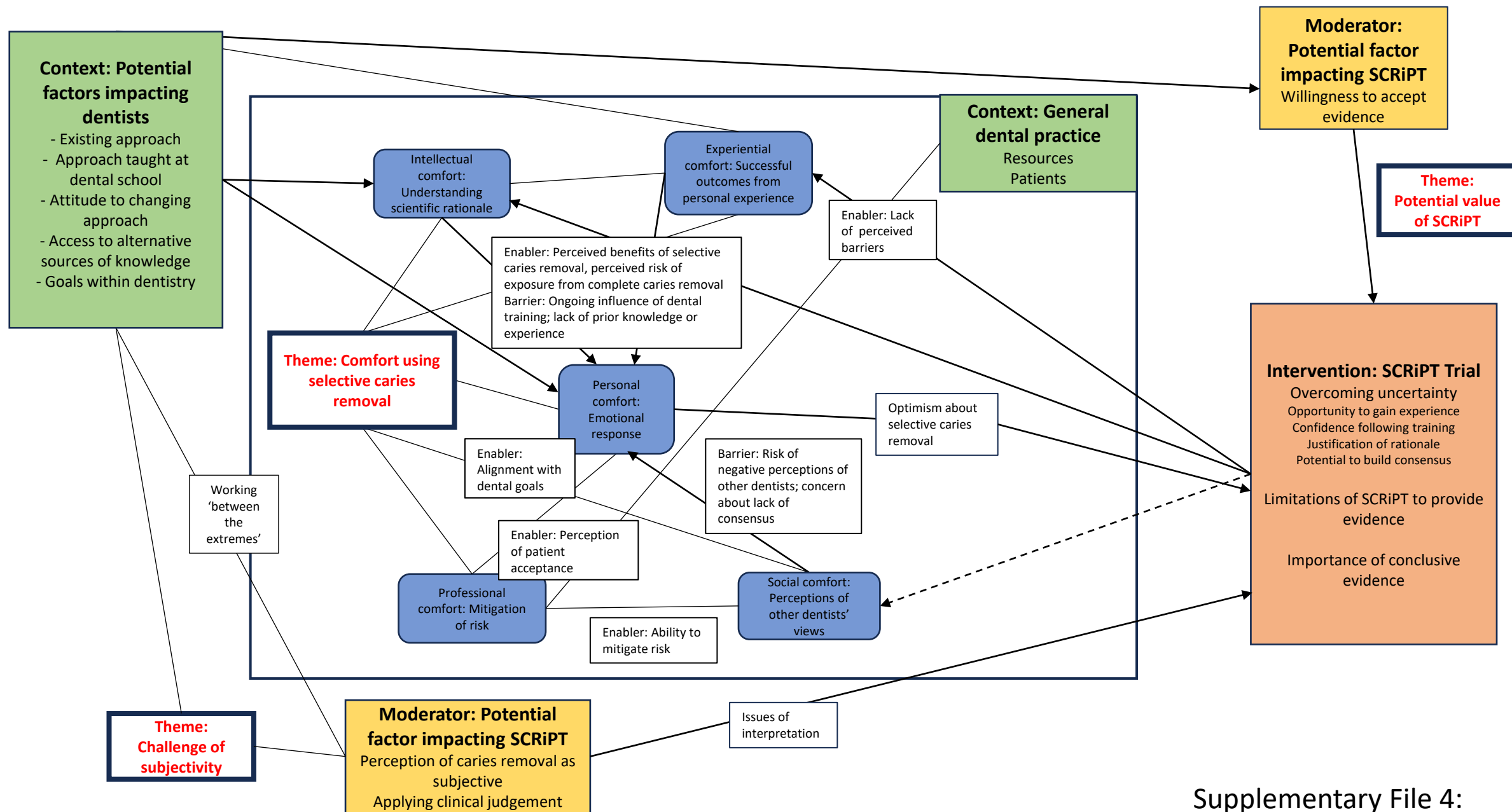

Supplementary File 4:  
Thematic map

Supplement: Supplementary file 4 — Thematic map. A figure mapping out the thematic framework. (.pdf) [file 12903_2025_5699_MOESM4_ESM.pdf]
